# “No generics, Doctor!” The perspective of general practitioners in two French regions

**DOI:** 10.1186/s12913-017-2682-5

**Published:** 2017-11-09

**Authors:** Béatrice Riner, Adèle Bussy, Jeannie Hélène-Pelage, Nycrees Moueza, Sébastien Lamy, Philippe Carrère

**Affiliations:** 1 Department of General Practice, University of the French West Indies, Pointe-à-Pitre, France; 2 Department of Clinical Pharmacology, Toulouse University Hospital, Toulouse, France; 3 Laboratory of Epidemiology and Analysis in Public Health, UMR1027 INSERM, University of Toulouse III Paul Sabatier, Toulouse, France; 4 Département de Médecine Générale, Faculté de Médecine Hyacinthe Bastaraud, Campus Universitaire de Fouillole, Université des Antilles, 97157 Pointe-à-Pitre Cedex, Guadeloupe, BP 250 France

**Keywords:** Primary health care, Drug substitution, Health expenditure, Health behavior

## Abstract

**Background:**

Generic medicines are essential to controlling health expenditures. Their market share is still small in France. The discourse and practices of prescribers may play a major role in their use. The purpose of this study was to explore the knowledge, attitudes and practices of general practitioners (GPs) toward generic medicines in two French regions with the lowest penetration rate of these products.

**Methods:**

An observational study was carried out from October 2015 to February 2016 in Guadeloupe and Martinique. The first qualitative phase involved a diversified sample of 14 GPs who underwent semi-structured interviews. The second phase involved a random sample of 316 GPs (response rate = 74%) who were administered a structured questionnaire developed from the results of the first phase.

**Results:**

Seventy-eight percent of the participants defined a generic drug as a drug containing an active substance identical to a brand-name drug, but only 11% considered generic drugs to be equivalent to brand-name drugs, and the same proportion believed that the generic drugs were of doubtful quality. The primary recognized advantage of generic medicines was their lower cost (82%). The main drawbacks cited were the variability of their presentation (44%), the confusion that they caused for some patients (47%), frequent allegations of adverse side effects (37%) and a lack of efficacy (24%), and frequent refusal by patients (26%). Seventy-four percent of the participants stated that they adapted their prescribing practices to the situation, and of this group, 47% prescribed the originator product simply on demand.

**Conclusion:**

Most surveyed GPs were not hostile towards generic medicines. They were caught between the requirements of health insurance regimes and the opposition of numerous users and suggested that the patient information provided by health authorities should be improved and that drug composition and packaging should be made uniform.

## Background

As populations get older and chronic diseases become more prevalent, countries with high or intermediate income levels have seen their health expenditures rise considerably during the last 25 years. For example, in the five main economies of the Euro zone (Germany, France, Italy, Spain, and Netherlands), total health expenditure increased from 1328 USD per capita on average in 1990 to 4322 USD in 2015 [1]. Outpatient pharmaceutical expenses were partly responsible for this increase. Among the five countries mentioned above, the expenditures increased from 214 to 604 USD per capita on average between 1990 and 2015 and comprised up to one-fifth of total expenditures [1]. It became necessary to implement policies that limited this growth in spending, and one of the levers for action was related to generic medicines. Since the late 1990s, most high- and middle-income countries have put in place legislations promoting the development of the generic market [2]. In the five main economies of the Euro zone, pharmaceutical expenditure increased faster than total health expenditure from 1990 to 2002, but since 2002, the situation has been reversed [1]. According to the IMS Institute for Healthcare Informatics, the existence of generic drugs saved up to 100 billion Euros in 2014 in Europe [3].

A generic medicinal product is defined by the European Medicines Agency as a “product that has the same qualitative and quantitative composition of active substances and the same pharmaceutical form as the reference medicinal product and whose bioequivalence with the reference medicinal product has been demonstrated by appropriate bioavailability studies” [4]. This definition guarantees that generic medications, or multi-source medications, adhere to the same quality norms as the originator products and that they are therapeutically interchangeable [5]. They are copies of products whose patent have expired, and they allow a considerable reduction in pharmaceutical costs. High-income countries have invested in this opportunity to varying degrees. In 2013, generic medicines represented 83% and 80% of the market volume of reimbursed medicines in the United Kingdom and Germany, respectively. In France, generics accounted for only 30% of the market volume [1]. The narrow repertoire of available generics, the impact of initial prescriptions, the circumvention strategies of the pharmaceutical industry, the critical positions taken by some scientific societies, and insufficient acceptability of generics by users have been identified as some of the barriers to the development of the market for generics [6, 7].

Through their prescribing choices and the trust that their patients have in them, physicians are may be able to play a greater role in the development of the generics market and the reduction of pharmaceutical costs. However, few research studies have examined the perception of generics by prescribers [8, 9], and the methodological quality of these studies is often open to criticism. Only two studies are available in France to the best of our knowledge. They were carried out in 2002 in central and south-eastern France using quantitative methods, and they obtained low response rates of 35% and 56%, respectively [10, 11].

Among the regions of France, Guadeloupe and Martinique – also called the “French West Indies” – have the lowest rates of penetration of generics on the pharmaceutical market. This penetration rate is defined as the volume share of generics sales among total sales of drugs eligible for substitution. This indicator is used to track the consumption of generics by department. In 2013, in the French West Indies, the penetration rate of generics was estimated at 73%, which is ten points below the national mean [12]. These regions have some specific aspects: their populations are predominantly of African or Indian descent, are younger on average and are more often in situations of poverty than populations in mainland France. However, the healthcare system in Guadeloupe and Martinique is identical to that developed on the national level. A study led in 2014 in the French West Indies showed that a quarter of users of the health insurance regime were hostile towards taking multi-source drugs, and notably, this opposition appeared to be related to the discourse and practices of outpatient GPs [13].

Our objective was thus to explore the knowledge, attitudes and practices towards generic medicines among GPs in Guadeloupe and Martinique. In particular, we aimed to assess definitions, perceived advantages, perceived drawbacks, prescribing practices, and proposals for improving the acceptability of generic medications in this population.

## Methods

We set up a cross-sectional study in Guadeloupe and Martinique from September 2015 to March 2016 using an exploratory design. The study was comprised of an initial qualitative phase with a small purposive sample of GPs, followed by a second phase with a large sample that involved quantitative analysis. This method has been previously used in the French West Indies [14].

### Populations

The source population was identified from the telephone directory (283 practitioners in Guadeloupe and 273 in Martinique in 2015). Inclusion criteria were a) office practice of general medicine and b) practice consisting at least partly of mainstream medicine.

The first phase of the study involved 14 GPs known to comply with the inclusion criteria. They were arbitrarily selected to constitute a diverse sample regarding age, gender and geographical area of practice.

**Fig. 1:**
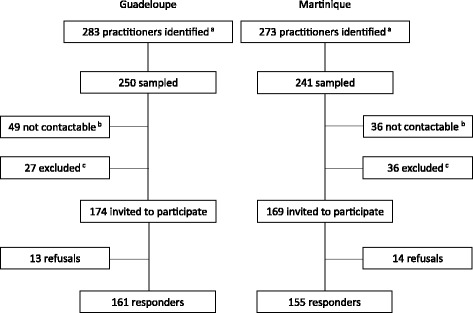
Recruitment of participants, second phase. *a* listed in the telephone directory. *b* at least 10 call attempts on different days and at different times. *c* practitioners with a specific mode of practice (exclusively ultrasonography, nutrition, acupuncture, homeopathy, mesotherapy or emergency medicine) and practitioners who are no longer practicing

The second phase involved a random sample of GPs. A minimum of 300 participants was expected, excluding those who participated in the first phase of the study. This sample size was calculated to restrict the margin of error to 3.5% with confidence level set at 5%. A first draw selected 198 practitioners in Guadeloupe and 191 in Martinique. Of them, 68 could not be contacted despite ten call attempts on different days and at different times, and 49 did not meet the inclusion criteria. Thus, a second draw was carried out among the remaining practitioners to reach the recruitment goal. In this second draw, 17 practitioners could not be contacted and 14 did not meet the inclusion criteria. Finally, 343 practitioners were invited, and more than 90% agreed to participate in the research. Taking into account the GPs who could not be contacted, the response rate can be estimated at 73.8%, i.e., 316 responders. This response rate represents almost two-thirds of the total theoretical number of GPs who work in ambulatory settings in Guadeloupe and Martinique and met the inclusion criteria. The recruitment process of participants in the second phase is summarized in Fig. 1.

### Data collection and definitions

In the first phase, data were collected by means of semi-structured interviews carried out face-to-face by two trained investigators. These interviews were based on four principal open-ended questions addressing knowledge, attitudes and prescribing practices about generics and proposals for improving their acceptability. The interviews were recorded and transcribed by each investigator, and all identifying information was removed to preserve anonymity. For quality control and validation, the transcripts were checked by each investigator.

Subsequently, the second phase was carried out using a structured questionnaire administered by telephone by the same investigators. The number of questions was kept to a minimum in order to yield the best possible response rate. The questions examined: a) gender, b) age, c) definition given of generic medicines, d) perceived advantages, e) perceived drawbacks, f) practitioners’ prescribing practices related to generics and f) proposals for improving the penetration rate of generics. The first phase allowed us to define the lists of possible answers, which were not brought to the attention of the second-phase participants. The investigators entered the participants’ spontaneous responses in real time on a computerized form according to the grid of pre-identified answers. A few second-phase responses were not in line with those identified in the first phase. They were collected in plain text and secondary coded after discussion between the investigators and the study coordinator.

### Analysis

In the first phase, in accordance with grounded theory techniques, data were analyzed by identifying key terms in the transcripts, grouping them by theme, and cross-checking all the transcripts. Both investigators coded all data independently to ensure reliability. Disagreements were resolved through discussion between investigators and the study coordinator. This analysis was carried out with N’Vivo v10 software.

In the second phase, data analysis was carried out with Stata v13 software. Responses were described as frequencies, percentages and 95% confidence intervals. The estimates were corrected using Stata “survey” commands to take in account the large sampling fraction.

The results presented here integrate the findings of the two phases of the study. For each topic, the second-phase quantitative results are clarified by excerpts from the transcripts obtained during the first phase.

## Results

The main characteristics of the studied populations are shown in Table 1. In the second phase, 37.7% of the responders were women and 55.0% were 55 years old and over. According to available medical demographic data [15], 35.4% of the GPs practicing in the French West Indies in 2015 were women and 53.2% were 55 years old and over. The distributions of gender and age did not differ significantly (*p* = 0.496 and *p* = 0.598, respectively).

**Table 1:** Characteristics of the participants

	First phase (*N* = 14)		Second phase (*N* = 316)	
	*N*	%	*N*	%
*Geographic location*				
Guadeloupe	*7*	50.0	*161*	50.9
Martinique	*7*	50.0	*155*	49.1
*Age, years*				
< 35	*2*	14.3	*18*	5.7
35 to 44	*4*	28.6	*39*	12.3
45 to 54	*5*	35.7	*85*	26.9
≥ 55	*3*	21.4	*174*	55.0
*Gender*				
Female	*7*	50.0	*119*	37.7

### Definition of generic medicines

Of the practitioners who participated in the second phase, 77.8% defined generic medicines as containing the same molecule or active substance as the originator medicine of which they are a copy (Table 2). In the first phase, in response to the question “How do you define a generic medicine?”, one practitioner answered: *“A generic medicine, as I tell my patients, is the same molecule, but it’s manufactured at a lower cost, so it’s less expensive and it’s more economical for the health insurance regime”* [M7]. For 25.3% of those surveyed, the lower cost of generic medications was an integral part of their definition. Differences in composition were nevertheless cited by 21.5% of the participants, and one practitioner stated the following: “[Generic medicines contain] *the same active substances as the original medication with excipients that can be different. They are considered to be equally effective at the same doses and in the same dosage forms as the original medicine”* [M4]*.* The notion of therapeutic equivalence was cited by 10.8% of the participants, but 11.1% stated from the outset that generics could be poor copies of the originator product.

**Table 2:** Definition of generic medicines

	*N*	%	95% CI
*How would you define generic medicines? (Several possible responses)*			
Same active substance as the originator	*246*	77.8	74.7–80.7
More economical	*80*	25.3	22.3–28.6
Different excipients	*68*	21.5	18.7–24.6
Poor-quality copy	*35*	11.1	9.0–13.6
Off-patent	*34*	10.8	8.7–13.2
Equivalent to the originator	*34*	10.8	8.7–13.2
Different presentation	*18*	5.7	4.2–7.6
Produced by a different laboratory than the originator	*17*	5.4	4.0–7.3
Efficacy equivalent to the originator	*13*	4.1	2.9–5.8
Name conforms with the INN^a^	*10*	3.2	2.1–4.7
Identical quality to the originator	*2*	0.6	0.3–1.6
Bioequivalent to the originator	*1*	0.3	0.0–1.1
Safety equivalent to the originator	*0*	0	0

^a^INN, International Non-proprietary Name

### Perceived advantages

For 81.6% of those surveyed, the principal attraction of multi-source medicines was the reduction of pharmaceutical expenditure (Table 3). Nearly one in ten responders appreciated the importance of this reduced cost in terms of the health insurance regime and the control of health spending*.* ln contrast, less than 2% of the respondents stated that generics could be advantageous to users.

**Table 3:** Perceived advantages and drawbacks

	*N*	%	95% CI
*What do you think are the advantages of generics? (Several possible responses)*			
Reduced cost of medicines	*258*	81.6	78.7–84.3
Advantages for the health insurance regime	*38*	12.0	9.9–14.6
Control of health expenditure	*29*	9.2	7.2–11.5
Coherence of prescribing by INN^a^	*18*	5.7	4.2–7.6
Advantages for the patients	*6*	1.9	1.1–3.2
Equivalent to the originator	*6*	1.9	1.1–3.2
Efficacy equivalent to the originator	*5*	1.6	0.9–2.8
Safety equivalent to the originator	*0*	0	0
Identical quality to the originator	*0*	0	0
*What do you think are the drawbacks of generics? (Several possible responses)*			
Patients may be confused by changes in presentation	*148*	46.8	43.2–50.5
Presentation and dosage form differ between laboratories for the same molecule	*139*	44.0	40.4–47.6
Patients report more adverse effects	*117*	37.0	33.6–40.6
Patients refuse generics	*81*	25.6	22.6–28.9
Patients report that generics are less effective	*75*	23.7	20.8–27.0
Personal doubt as to equivalence	*66*	20.9	18.1–24.0
Poorer treatment compliance by patients	*63*	19.9	17.2–23.0
Complexity of prescribing by INN ^a^/name is difficult for the patient	*52*	16.5	13.9–19.3
Excipients differ between generics and from the originator	*42*	13.3	11.0–15.9
Personal doubts as to efficacy	*35*	11.1	9.0–13.6
Doubtful quality	*32*	10.1	8.1–11.5
Negative impact on the physician-patient relationship	*28*	8.9	7.0–11.1
More frequent allergic manifestations	*25*	7.9	6.2–10.1
Personal doubt as to safety of generics	*23*	7.3	5.6–9.4
Doubtful manufacturing origin	*20*	6.3	4.8–8.3
Complexity of prescribing by INN ^a^/name is difficult for the practitioner	*18*	5.7	4.2–7.6
Generics infringe freedom of prescription	*13*	4.1	2.9–5.8
Unacceptable demand by the state or health insurance regime	*11*	3.5	2.4–5.1
Poor presentation of generics compared with originators	*7*	2.2	1.4–3.6

^a^INN, International Non-proprietary Name

### Perceived drawbacks

The main reported disadvantage (Table 3) concerned differences in the presentation and dosage form of generic medicines between manufacturers (44% of the questioned practitioners). These differences were a source of confusion for the patients (46.8% of the practitioners), and some participants suggested that this variability in presentation put patients at risk. For example, one GP said the following in the first phase: “*When they are used to taking their little green tablet (…) and then the color of the tablet changes, they take the old tablet as well as the new one”* [G2]. The difference in presentation altered the placebo effect of some brand names: *“The placebo effect of a treatment can sometimes be a good thing. Doliprane will always be Doliprane, whereas paracetamol doesn’t work for some people”* [G1]*.* Substitution affected patients’ trust, particularly among the most elderly: *“Because after all, they’ve been used to their treatment for 30 years (…). Why change when you’ve got something that works?”* [M5]. The image that users have of generic medications is still marked by negative a priori attitudes due to their lower cost: “*Generics are presented as cheaper medicines (…), so the patients think they’re not being treated as well”* [M1]; *“they don’t want them because they think they’re inferior medicines”* [G5]. More than a third (37%) of surveyed practitioners declared that adverse effects were more frequently reported by patients taking generics. Some first-phase participants noticed the existence of specific adverse reactions: *“I see side effects. Some patients exaggerate them, but there are real side effects, and they aren’t the same in generics as in the originator”* [G7]*.* Finally, 25.6% of the participants stated that they were regularly faced with users who refused to take generics. For 19.9%, this refusal compromised treatment compliance.

One in five of the surveyed practitioners (20.9%) had personal doubts as to the equivalence between generic and originator products. These doubts seemed to mainly relate to differences in the excipients. For example, during the first phase, one practitioner stated: *“The* [generic] *medicine has been produced from an active substance, but* [with a] *coating that is different from the original (…). Therefore, the active molecule is not necessarily assimilated in the same conditions as the originator medicine”* [M2]*.* These doubts also arise from a lack of information on the changes in the composition of generics: *“The laboratories just simply changed the excipient as they liked, without warning (…). In the end, we don’t know what the generic medicine is made of”* [G2]. The diversity of generic laboratories was questioned: *“Do ten laboratories supply an identical medicine at an identical dose? Is the efficacy identical?”* [M2].

In the second phase, one participant in ten (8.9%) emphasized the negative impact of generic medications on the quality of the physician-patient relationship. As semi-structured interviews make it possible to follow up on a topic, this negative impact appeared more clearly in the first phase: nearly all participants in this phase expressed difficulties with substitution to varying degrees, particularly because of the time taken up by negotiating substitution with the users. One practitioner stated: *“During a day spent seeing patients when we work from 7 o’clock in the morning until 9 o’clock in the evening, we’re sure to have one hour of discussion about generics. That’s one hour lost in terms of quality of care”* [M2]. Negotiations with refractory users sometimes resulted in great lassitude: “*I’m tired, I don’t want to spend three hours arguing”* [M6]*.* These difficulties could be violently experienced: *“It’s getting difficult to practice medicine in these conditions. One of these days, it’s going to end badly, a doctor’s going to be punched”* [G6]. The perceived demands of the health insurance regime were felt severely: *“I’m going to have financial penalties if I don’t prescribe generics”* [M2]*.* Distress was expressed: *“We are always struggling (…). On the one hand, we’ve got patients who don’t want them, on the other [hand], we’ve got the health insurance regime who is putting on the pressure, and we’re in the middle”* [G5].

### Prescribing practices

In the second phase, one participant in ten stated that they only prescribed originator medicines (Table 4). The large majority (73.7%) of practitioners surveyed said that they adapted their prescribing practices according to the situation. Of the latter, 47.2% (34.8% of the total sample) declared that they prescribed originators when their patients asked for them. In the first phase, some explanations were obtained. Certain practitioners seemed simply to integrate the users’ expectations: “[Patients] *don’t understand why we can’t give them access to the medicines that are in front of them, are available at the chemists and have always done them good, and now we propose something else”* [M2]. The singular nature of physician-patient relationships may also intervene: *“When I do a home visit to a woman who always offers me something to drink, it’s difficult to say to her, ‘Today, I’m going to give you generics’, when she had said to me, ‘I don’t want them’”* [G4]. To avert negotiation and conflict, offending the patient was avoided: *“I’m not going to quarrel with a patient over a question of generics, I’ve got other things to do”* [M3].

**Table 4:** Prescribing practices

	*N*	%	95% CI
*In your routine practice, what are your prescribing choices in regard to generics or originators? (Only one possible answer)*			
It depends on the situation	*233*	73.7	70.7–76.6
I prescribe only generics or by INN ^a^	*50*	15.8	13.5–18.4
I prescribe only originators	*33*	10.4	8.4–12.9
*You answered, “It depends on the situation”. Can you expand on this response? (Several possible answers)*			
I prescribe an originator if the patient asks for it, even without a reason	*110*	34.8	31.4–38.3
A priori, I systematically prescribe an originator for certain drugs	*89*	28.2	25.0–31.5
A priori, I systematically prescribe an originator for fragile patients	*61*	19.3	16.6–22.3
I prescribe an originator on a case-by-case basis if the patient reports adverse effects	*50*	15.8	13.4–18.6
I mainly prescribe by INN ^a^	*36*	11.4	9.3–13.9
A priori, I systematically prescribe an originator for certain diseases	*31*	9.8	7.9–12.2
I prescribe an originator on a case-by-case basis if I observe adverse effects	*22*	7.0	5.3–9.0
I prescribe by INN ^a^ with the name of the originator in brackets	*22*	7.0	5.3–9.0
I prescribe by INN ^a^ or a generic as the initial prescription	*10*	3.2	2.1–4.7
I prescribe an originator on a case-by-case basis if the patient says that the generic was not effective	*8*	2.5	1.6–4.0
I prescribe an originator on a case-by-case basis if I see that the generic was not effective	*7*	2.2	1.4–3.6

^a^INN, International Non-proprietary Name

### Proposals for improving the acceptability of generic medications

The first measure proposed (Table 5) to facilitate the prescription of generics was related to improving the information given to users by the authorities (33.2% of the surveyed practitioners) rather than information provided by the practitioners themselves (13.9%). One practitioner stated: *“The health service regime ran a campaign about antibiotics, [and] they ought to run one about generics”* [M5] and another clarified by adding: *“to explain that they’re just as effective”* [M4]. The second measure most often cited (26.9% of those surveyed) related to standardization of the presentations and dosage forms of the pharmaceutical products. The third measure (14.2% of the participants) concerned the need to harmonize the communication and practices of health professionals. Above all, communication between practitioners and pharmacists needs improvement: *“The pharmacists pass the buck back to the doctors* [by saying] *‘If you don’t want a generic, you’ll have to pay for it, or else go and see your doctor* [to get the prescription changed]*!’”* [M4]. However, practices between physicians are also inconsistent: “[The patient] *doesn’t understand why his family doctor don’t write out exactly the same prescription as the hospital doctor (…). That leads to more trouble”* [M2]. Nearly one in ten practitioners proposed that brand names should be dropped and that systematic initial prescribing by International Non-proprietary Name (INN) should be adopted. The same proportion stated that they lacked convincing evidence of the equivalence between generics and originators, and some practitioners seemed to want better support: *“They ought to have given us training beforehand, we’d have more arguments to defend ourselves”* [M5].

**Table 5:** Proposals for improving the acceptability of generic medicines

	*N*	%	95% CI
*What measures do you think could make it easier to prescribe generics and make them more acceptable? (Several possible answers)*			
Public education and information campaigns for patients	*105*	33.2	29.9–36.7
Identical presentation and dosage form for generics and originators	*85*	26.9	23.8–30.2
Harmonization of communication between practitioners and pharmacists	*45*	14.2	11.9–17.0
More information given to the patient by the physician	*44*	13.9	11.6–16.6
More studies demonstrating the equivalence of generics and originators	*35*	11.1	9.0–13.6
Abandonment of brand names	*33*	10.4	8.4–12.9
Initial prescription by INN ^a^	*30*	9.5	7.6–11.8
Financial penalties for patients	*26*	8.2	6.4–10.5
More transparency about the manufacturing process of generics	*24*	7.6	5.9–9.8
More transparency about the manufacturing origins of generics	*20*	6.3	4.8–8.3
Education and information campaigns aimed at physicians	*17*	5.4	4.0–7.3
Harmonization of communication between general practitioners and other specialists	*12*	3.8	2.6–5.5
Financial penalties for physicians	*5*	1.6	0.9–2.8
Financial incentives for patients	*1*	0.3	0.0–1.1
Financial incentives for physicians	*0*	0	0

^a^INN, International Non-proprietary Name

## Discussion

In this representative sample of GPs working in outpatient settings in Guadeloupe and Martinique, the definition given to generic medicines was primarily related to the notion of low-cost copies. Equivalence was mentioned by only one in ten practitioners. The precise concept of bioequivalence, which is fundamental to justifying therapeutic equivalence, was hardly cited. Previous studies [16, 17] emphasized the lack of knowledge about generics among primary care physicians. Previous studies also showed that many practitioners feel that the generics-originator equivalence has not been sufficiently demonstrated [18–20]. However, in countries which have adopted international norms of pharmaceutical production control, an increasing number of studies nevertheless attest to clinical equivalence [21, 22].

A large proportion of our sample expressed fears about the harmful effects of generics, in particular because of the variability of their excipients and presentations. In other countries, differences in name, form and color of the products are also perceived by prescribers as creating a risk of confusion for users [20, 23, 24]. Studies in other countries have also shown that differences in the use of excipients between one generic laboratory and another affect practitioners’ confidence in the conformity of generics with originators [16, 24].

The proportion of participants in our study who expressed doubts about the efficacy and safety of generic medications was consistent with data in the literature. According to the meta-analysis carried out by Colgan et al., which included 17 studies performed in 12 countries, a quarter of the medical population between 1987 and 2015 considered generic medications to be less effective and less safe than their originators, and there was no significant change in this proportion over time [8]. Beyond the practitioners’ lack of knowledge and the generics’ variability, several authors have suggested that these negative attitudes result from pressure from the pharmaceutical industry on professionals [9, 11, 23, 25]. They may also be a matter of pressure from patients.

According to the meta-analysis of Colgan et al. on 27 studies carried out between 1990 and 2015 in 16 countries, one-third of the general population doubted the efficacy of generics and one-fifth doubted their safety [8]. In 2014 in the French Indies, 46% of a sample of users of the health insurance regime doubted that generics were effective and 36% doubted they were safe, whereas 17% stated that they had experienced inefficacy and 18% stated they had experienced adverse effects related to taking these medicines [13]. Generics are marked by unfavorable social norms, which are strongly expressed by the users. Widely held constructs regarding relationships between a product’s quality and its cost play a role [26, 27]. Generics lose the symbolic value of branded drugs through their name and presentation changes; these changes can impact users’ perception of curative function [28] and safety of use [29]. Furthermore, according to Sarradon-Eck et al., negative representations of generics can result from an opposition or resistance to administrative power, which is increasingly evident in the field of health [28]. This phenomenon could occur in prescribers.

A large proportion of the surveyed practitioners prescribed originator medicines at the patients’ request, without discussion. This practice was rarely described in the literature [24, 30]. In our sample, it seems to be an avoidance behavior to cope with the pressure from patients and the negotiations that the conversations entailed. The increased length of the consultations due to such negotiations, which has already been highlighted [20, 31], was poorly tolerated. The impact on the quality of the physician-patient relationship appeared to be so negative that it was experienced with difficulty or even violence by some of the surveyed practitioners. These practitioners highlighted the responsibility of the authorities, criticizing a lack of information for patients. In a report edited in 2014 [32], the French Court of Auditors advocated an increase in communication campaigns dedicated to users. A lack of support from practitioners was also mentioned, which was confirmed by their lack of knowledge about generics. More fundamentally, some surveyed physicians did not seem to adhere to their assigned role regarding the development of the generics market. It is known that many GPs do not wish to exercise the economic accountability that the authorities want them to endorse [33], especially when they feel that it could contravene their own professional ethical rules [34].

One of the strengths of this study is the method that was used. It made it possible to both quantify and understand the examined elements. Moreover, the response rate achieved in the second phase can be considered high, given the target population and compared to previous studies. A limitation of this study is the recruitment procedure used in the first phase. The recruitment number was set a priori at 14 practitioners. However, the second phase allowed us to obtain response saturation to the main issues addressed here. A second limitation is the data collection procedure used in the second phase. Interviews were not recorded at this phase because of the opposition of most responders and regulatory limits; thus, the quality of completion of the computerized form could not be checked. Beyond misunderstandings or random errors, the subjectivity of the investigators could have induced biases, the global effects of which are difficult to predict. However, these data collection procedures had the major benefit of not influencing participants’ responses.

## Conclusions

In conclusion, the great majority of GPs are not opposed to generic medicines. However, they must face the hostility of numerous users. Many physicians lack the data demonstrating equivalence between generics and originators and have difficulties convincing the most distrustful patients. These challenges can have a negative impact on their practice conditions. The lack of involvement of some GPs in their health-cost regulation mission could also be an obstacle in the development of the generics market.

In France, policies to develop the generics market were initially designed from a top-down approach [35], and the views of the prescribers may have not been sufficiently taken into account. This study allowed us to present some GPs’ proposals to improve the acceptability of generics. Users require better information from authorities. This information cannot be restricted to demonstrations of the economic interest of generics; it must convince users of their safety and quality. Practitioners need better support from health insurance regimes and better cooperation with other health professionals to avoid inconsistencies in communication and practices. In addition to systematically prescribing drugs by International Non-proprietary Name, one of the simplest solutions to make generics more acceptable to both prescribers and patients could be uniformization of their presentations by delivering exact copies (same active and inactive ingredients) or the same generic product to the same patient for a given originator product.

## Abbreviations

95% CI: 95% confidence interval
INN: International Non-proprietary Name
